# Use of Magnetomechanical Effect for Energy Harvesting and Data Transfer

**DOI:** 10.3390/s22093304

**Published:** 2022-04-26

**Authors:** Rafał Mech, Przemysław Wiewiórski, Karol Wachtarczyk

**Affiliations:** Faculty of Mechanical Engineering, Wroclaw University of Science and Technology, 50-370 Wroclaw, Poland; przemyslaw.wiewiorski@pwr.edu.pl (P.W.); karol.wachtarczyk@pwr.edu.pl (K.W.)

**Keywords:** smart materials, magnetostriction, Terfenol-D, wireless sensors, ultrasonic system

## Abstract

The presented paper describes a method where, with the use of a dedicated SMART Ultrasonic Resonant Power System (SURPS) developed by the authors, a power and data transfer between two devices can be performed at the same time. The proposed solution allows power to be supplied to the sensor, located in a hardly accessible place, with simultaneous data transfer in a half-duplex way (e.g., “question–response”). The power transmission mechanism is based on the excitation of a construction with a sinusoidal wave, with an actuator transforming this wave into useful, electrical power through a harvester device. Data transfer is achieved with the use of the F2F (Frequency Double Frequency) procedure, which is a kind of frequency modulation. To receive optimized parameters for each construction, an original software is developed, which allows the selection of the proper type of actuator, modulation, and frequency.

## 1. Introduction

In the past few decades, the development of wearable and wireless devices has been growing significantly. It became possible to reduce the power which is needed to supply these devices to only tens of milliwatts [1]. At those power levels, traditional batteries are limited to only short-term operation, mainly due to dimension limitations. Additionally, in the case of long-term operation, batteries need to be replaced or recharged while, at the same time, undergoing degradation. Meanwhile, other components behind wearable and portable devices improved rapidly following Moore’s law [2]. To overcome the problem of traditional batteries, researchers started to work on energy harvesting. This is a technique that can extract electrical power from ambient sources and might supplement and even replace batteries.

Energy Harvesting (EH), originally known as power harvesting or energy scavenging, is a set of techniques that provide electrical energy through energy conversion from different sources, such as mechanical, thermal, solar, and electromagnetic energy and salinity gradients, etc., e.g., [3]. Generally, the main goal is to use sources that are commonly available in the environment, which, in most cases, are undesirable and suppressed (e.g., noise, impact, and mechanical vibration from equipment and constructions and different sources of heat from friction or combustion or as a result of electric current flow and engine cooling, etc.). Energy harvesting is also based on commonly available energy sources (solar light, wave energy, salinity differences, and biochemical processes, e.g., plants), as well as on energy connected with human biology (motion, body heat, etc.). Nowadays, it is said that EH might be a useful source of “low-cost or cost-free” (excluding installation costs) power supply to low-power electric devices [4,5,6,7,8]. Currently, many types of research are being carried out in relation to vast energy harvester networks which provide a relatively large amount of energy in a short time.

One of the sources of wasted energy is structural vibrations. In many cases, it can be a consistent source of energy, even though its amplitude and frequency can vary significantly, depending on the location. Vibrations in civil engineering constructions, such as buildings or bridges, have low amplitudes and frequencies (0.1 g and 0.1 Hz); at the same time, various small electrical devices, such as ovens, microwaves, and others, have higher amplitudes and frequencies (0.5 g and about 150 Hz, respectively) [9]. In other constructions, such as cars, planes, or helicopters, vibration amplitudes are relatively high, while amplitudes are varied dependent on operation conditions [10,11]. The above conditions have inspired multiple types of harvester to be developed and described in the literature.

Two types of harvester device can be distinguished, i.e., passive materials and active materials. Those which are passive-type harvesters can be divided into electromagnetic and electrostatic. The electromagnetic devices use Faraday’s law, and they are built from a coil and permanent magnet. The relative motion of these two elements generates AC voltage [12,13]. The electrostatic devices are variable capacitors with movable electrodes and a dielectric layer between them. The motion of the layers caused by the vibrations induces AC currents [14,15,16,17]. In the case of active harvesters, most devices are based on magnetostrictive or piezoelectric (PZT) materials [18,19]. At this point, it should be noted that piezoelectric harvesters are capacitive sources of energy; therefore, they have a high output impedance. This implies that appropriate energy management circuits must be used to be able to supply electrical devices. On the other hand, there are magnetostrictive harvesters, which are inductive. Thus, they can provide low impedance at frequencies characteristic of most common sources of vibrations (described above).

Of the passive vibration energy harvesters, magnetostrictive harvesters supply higher energy density. What is more, a comparison of magnetostrictive devices with those based on piezoelectric material showed that both can generate similar levels of output energy; however, there is no need for additional special power management circuits in the case of solutions based on magnetostrictive material. Among the magnetostrictive devices, the two most common types are the axial type and bending type, based on the stress state in the material. The axial-type devices are usually mounted in places where a large excitation force is provided [20,21,22,23,24,25,26,27,28]. Because of these high loads, they can generate relatively high power density levels up to even 10 W/cm^−3^ [23]. However, to protect core material from damage, proper mechanisms of protection are needed, especially in the case of brittle Terfenol-D [23,24]. Contrary to axial-type harvesters, the bending type of vibration energy harvesting device can be mounted on any source of vibrations [29,30,31,32,33]. However, the output power density is much lower, and the typical level is 10 mW/cm^−3^ [30,32]. Additionally, bending harvesters can be divided into three different types: a single-layer magnetostrictive beam, the output of which is relatively small [29,30]; a double layer, the output of which is greater than for single layer [34] but is still relatively small, mainly because of dominant shear stresses; and a composite magnetostrictive beam [31,32], which provides the highest amount of energy of these three types but requires further investigations. It should be noted that, in the case of magnetostrictive harvesters, their efficiency varies and depends on many factors, such as load, frequency of operation, method of mounting, or the material on which the given device is based. In study [32], it was seen that the efficiency of the energy conversion of the proposed device was 16% at 395 Hz; however, in [31], the maximum conversion efficiency was 35%, and it was achieved at an input frequency of 202 Hz.

In the literature on the subject, it can be seen that the research focused mainly on piezoelectric transducers [5,6,7,35,36]. However, it turns out that, in some cases, a better solution is the use of magnetostrictive harvesters [37]. Taking into account the previous research conducted in this area, the main goals of this research are:

- development of a system that allows the transmission of energy and information through a solid in the case of ultrasonic frequencies (the system should operate at frequencies above 20 kHz, i.e., inaudible to most people);
- use of smart materials (piezoelectric and magnetostrictive);
- development of the test stands which allow the determination of the operating parameters of the device.

In this work, the axial-type harvester is presented. Such a harvester was chosen after analysis of already developed harvesters, which can be found in the literature. Additionally, such a solution was also connected with the predicted amount of energy which might be generated with the use of this type of harvester. It can be seen, based on the literature research, that higher amounts of energy can be obtained from axial-type harvesters.

## 2. Materials and Methods

The results presented in the paper are related to the magnetomechanical effect. The main material used in the research was Terfenol-D (a material with so-called giant magnetostriction). Additionally, the research used material prepared by the authors, which was produced by the suction casting method.

The prepared material was Fe_57_Co_10_B_20_Si_5_Nb_4_V_4_, which is given using atomic notation. The elements used for the preparation of the alloy were melted into uniform material with the use of a laboratory furnace. High accuracy of chemical composition was obtained by using elements of very high purity and using a scale with high accuracy. The weighted elements were mixed and placed in the furnace to create alloy material. After melting the elements three times in the argon atmosphere, the obtained alloy was again heated to the temperature of the liquid and then rapidly sucked into the copper mold, which was constantly water-cooled. The materials prepared with this method were in form of rods.

To achieve the main goal set by the authors, the design of a multiphase magnetostrictive actuator was developed. This device served as an actuator of a multiphase mechanical vibration regulator, which allowed the positioning of objects that are freely placed on the beam. Positioning was performed using mechanical vibrations. It was assumed that the proposed solution will make it possible to supply energy in a controlled manner and remotely perform mechanical work (e.g., displacement in a given direction, rotation of a mechanism, unscrewing an element by pressing a switch). The described process should be possible to carry out with the use of vibrations up to 30 kHz. To accomplish this task with the use of magnetostrictive actuators, the idea of generating phase-shifted vibrations that have the same frequency was implemented. The implementation of this idea consisted of switching on the actuators, which were placed one after the other, and the signals sent by them were shifted relative to each other by an appropriate angle, which was controlled by the developed algorithm. The whole system used feedback obtained from a vibration sensor, thanks to which the presented method allowed the use of the so-called Structural Stiffness Code (CSS).

### 2.1. Magnetostrictive Actuators

To be able to generate mechanical vibrations, first, it is necessary to understand the operation of the different types of actuator. The selection of an actuator for a given task depends on the vibration level (PSD—Power Spectral Density), the operating frequency, and the level of amplitude-phase distortions. When we consider devices without moving elements, i.e., solid-state devices, we limit the choice between two types of transducer, namely, piezoelectric (PZT) and magnetostrictive (usually based on Terfenol-D). In the case of piezoelectric devices, it should be noted that they only work in a strictly defined range of resonance frequencies. This range is related to the design of the transducer. In the case of the operation of such devices outside their scope, the actuator overheats, and the ceramic material is usually destroyed. In the case of actuators based on a magnetostrictive material, the frequency range in which they can operate is much wider compared to PZT actuators. The operating frequency of the magnetostrictive actuator can reach 100 kHz [38] and also includes the resonant frequencies of the actuator itself [39]. Additionally, such actuators generate large forces, which, depending on the design and size of the device, can reach up to several hundred kN. Despite the above advantages, these actuators also have some drawbacks, the most important of which are the non-linear operating characteristics, low vibration amplitude, and the limited operating temperature associated with the use of an induction coil. Additionally, the price of such actuators is relatively high when compared to devices based on PZT. However, due to the abovementioned advantages, it was decided to use magnetostrictive actuators in the solution presented in this paper.

### 2.2. Magnetostrictive Harvesters

In the case of magnetostrictive harvester actuators, the most important element is the magnetostrictive core. Such a core may consist of one or more elements, depending on the size, length, and purpose of the device. The material from which the core is made is also an important issue. Such material must be characterized by gigantic magnetostriction (GMM—Giant Magnetostrictive Materials, e.g., Terfenol-D, nano-ferrite cobalt). An additional element is a system that allows you to adjust the pre-magnetization of the material, which are usually properly selected neodymium magnets. The number of elements that make up the core of the device has a significant impact on the frequency of operation. The smaller the number of elements, the higher the operating frequency can be. To optimize the construction of the actuator, the magnetostrictive material and neodymium magnets are arranged alternately.

In the case of magnetostrictive materials, the parameters of the physical fields that affect the material from the outside, i.e., the magnetic field and also the mechanical field, are of great importance. Therefore, to obtain the best working parameters, permanent magnets and appropriate spring systems are used. The springs are used to create initial stress in the magnetostrictive material, the appropriate level of which makes it possible to increase the amplitude of work. In addition, the main task of the permanent magnets is to shift the starting point of the material’s operation, thanks to which such devices can work in the linear range of their characteristics.

The actuators/harvesters presented in this paper have relatively large dimensions: the diameter of the device was 44 mm and its height was 47 mm. The geometry was forced mainly by the necessity of applying the appropriate actuator pressure to the tested structure. Inside the casing was a coil with a resistance R_coil_ = 5.5 Ω. The devices worked in a wide frequency range from 10 Hz to 30 kHz to find the resonant frequency of the system within which the system obtained the highest voltage values. Additionally, both harvesters and actuators were pre-stressed with a force of 400 N. This value was determined based on experimental tests, during which the magnetomechanical response of the system depending on the applied load was determined.

**Figure 1:**
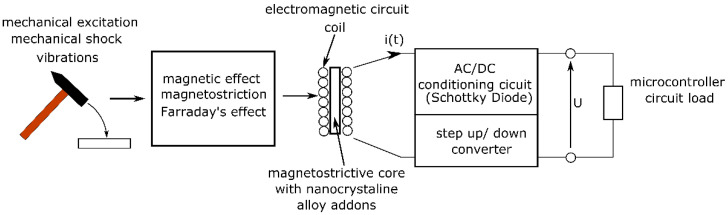
Harvester scheme.

The above information is based on the authors’ extensive experience in the construction and modernization of magnetostrictive actuators [38]. Subsequent constructions and modifications allow more and more power to be obtained; therefore, these devices began to be used as a source of electricity (Figure 1). The amount of electricity, i.e., the values of current and voltage, must be appropriate for the power supply of the sensor and the built-in processor (with a matched converter) and the communication system. In addition to these parameters, the generator voltage/current conditioning system is equally important. Additionally, to properly design the harvester’s electrical circuits, it is necessary to know the characteristics of the receiving device.

### 2.3. Electric Transducer

Harvesters can be divided into direct current and alternating current. DC harvesters include devices that use the thermoelectric or photovoltaic effect. On the other hand, AC harvesters are devices that generate energy from vibrations (magnetostrictive, piezoelectric, and the Faraday effect). Harvesters that use a mechanical impulse (shock) to generate energy are a special case [38]. In the event of an impact, electricity is produced and available for a very short time. Impact harvesters are characterized by a strong current pulse that appears in the form of an alternating current. At the same time, in the generated signal, one can observe the frequencies related to the magnetic resonance between the core and the coil of the device. Harvesters of this type differ in the amount of magnetostrictive material and their form (solid, composite, powdered) in the magnetic circuit. The power that can be obtained from this type of harvester depends on the type of material, layout, and dimensions of the core.

## 3. Results

### 3.1. Multiphase Actuator

Mechanical vibrations in a wide frequency range were generated with the use of a multiphase magnetostrictive actuator. The system also had an integrated sensory part. The control of the system was carried out using the HERON Advanced Multiphase software. This software allowed us to supply the actuator with control signals, which kept the whole structure in resonance. The vibration controller used an electronic system containing DSP (Digital Signal Processor) and measurement modules, i.e., input–output modules, DDS (Direct Digital Synthesis) generators, and an ICP (Integrated Electronics Piezoelectric) sensor from PCB Piezotronics. A dedicated CDM-P1 device was responsible for conditioning the signal and changing the vibration amplitude.

**Figure 2:**
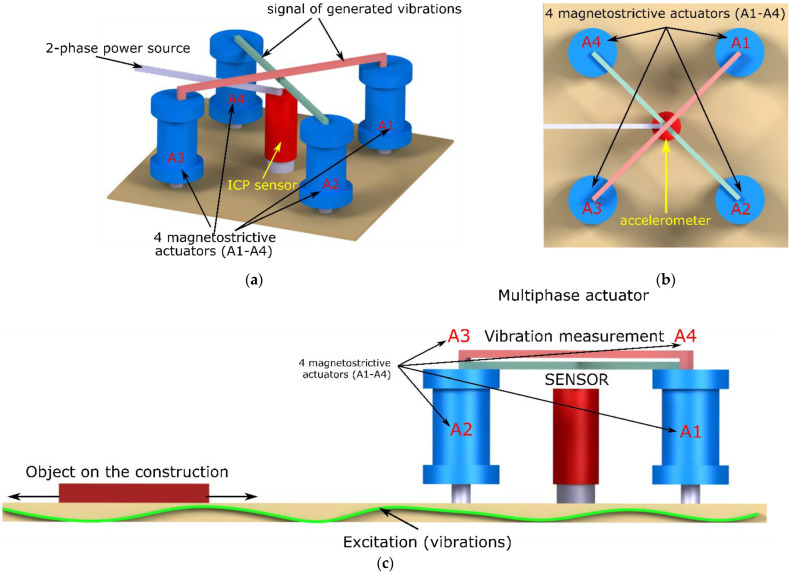
The idea of the four magnetostrictive actuators (A1–A4) system: (**a**) actuators operating simultaneously with vibration measurement path; (**b**) symmetrically distributed actuators; (**c**) linear positioning system using vibration.

To generate the so-called multiphase vibrations, it is necessary to use a head that contains many magnetostrictive actuators. The research presented in the paper was carried out with the use of a head consisting of four actuators equipped with Terfenol-D cores and a nanocrystalline alloy prepared by the authors. Such an arrangement of actuators allowed for their analog control similar to that of a typical stepper motor. In addition, a PCB-type vibration sensor, which was located at the central point of the system, was used to measure the vibration values in the tested object. A diagram of the arrangement of actuators with the PCB sensor is presented in Figure 2.

The prototype head was manufactured according to the design shown in Figure 2. The actuators were arranged symmetrically (Figure 2b). In addition, Figure 2c schematically shows the positioning system that used the generated vibrations for a straight rail. This circuit worked with a feedback loop, which is described below.

**Figure 3:**
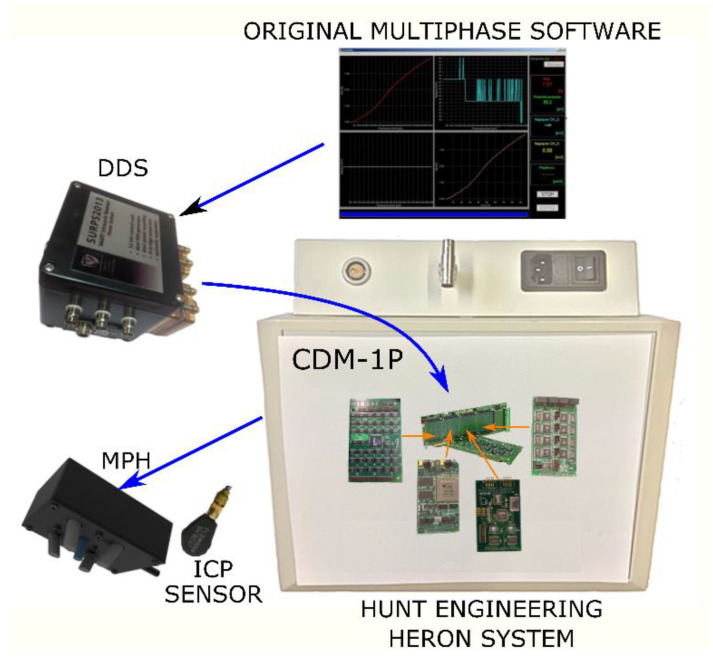
Elements of the control system based on Hunt Engineering DSP.

Figure 3 shows the vibration control system with the designed head. Importantly, in the case of this system, it was possible to control each actuator separately, thanks to which it was possible to generate even very complex mechanical vibrations. In addition to the designed head, the vibration controller consisted of many advanced electronic systems based on the HERON card with a floating-point DSP and the Texas Instruments C6000 card with expansion modules. Moreover, the system was supplemented with dedicated software using API.

The Hunt Engineering HERON system was responsible for signal processing in the system. Thanks to the use of this system, it was possible to comprehensively design the experiment, because the software allowed for the acquisition and conditioning of the sensor signal and the generation of phase-shifted control signals. For this purpose, both a DAC (Digital-to-Analog Converter) and a DDS were used.

The PCB sensor received vibrations in the form of electrical signals and then, after being appropriately supplied, they were collected by a module of 16-bit ADCs-HEGD12. They were used as the basis for the determination of the setpoints of the digital vibration controller which released subsequent DDS values in the feedback loop through HEGD4. The role of CDM-1P was only the conditioning of sensor signals and power amplification for the four magnetostrictive actuators.

The HERON Advanced Multiphase software used in the system was applied to maintain the structure in resonance, although the resonant frequencies were changing. To achieve such an effect, the system generated a control signal for actuators with the appropriate frequency of resonant vibration. Determining the appropriate resonant frequency was possible based on the analysis of the signal with a given moment of frequency. In the case of a decrease in the vibration amplitude at a given excitation, a deviation from the resonance state was found. In the next step, the system checked how the system would behave in the case of excitation with a higher and lower frequency. If, in any of the cases, the amplitude of the vibrations increased, then the system defined this frequency as the new resonant frequency. In the next step, the operation of checking the amplitude value was repeated.

### 3.2. Remote Object Positioning

One of the tasks that were assigned to the designed system was the positioning of the object on the structure with the use of vibrations. To carry out this task, in the first stage, it was necessary to generate vibrations with a resonance frequency in the structure. Then, a non-magnetic object weighing about 30 g was placed on the vibrating structure (a steel beam 6 m long). The vibrations caused by the beam set the mass in motion—the element was jumping on the beam and hitting it. These impacts were used to determine its position on the object through changes in the phase shifts in the generated signals.

Due to the application of the acceleration sensor in the developed multiphase head, it was possible to register the acoustic events that occurred when the mass separated from the beam. Due to the small mass of the object (20 g), the frequency of the system only slightly changed (0.04%). In addition, the previously described acoustic event related to the separation of the mass from the beam had a much higher frequency than the resonant frequency of the beam itself. The changes in vibrations were recorded as a sinusoidal signal with a frequency of 667 Hz.

### 3.3. Code of the Structure Stiffness—CSS

The above-described acoustic event that occurred in the system during the impact of the mass against the beam was characteristic of each structure. In the event of a change of mass or construction change, the response changed. It was related to the different amounts of energy accumulated over time. This response served as a control signal for the vibration controller. In addition, the ability to control the amplitude of vibrations and measure the response of the structure made it possible to detect changes in it, including the appearance of defects or damage.

Based on these tests, it can be concluded that the number and nature of recorded acoustic events are mainly influenced by the energy (in the form of mechanical vibrations) supplied to the tested structure and the temporary state of the structure in which they are located. Hence, the sequence of acoustic events as a modulated binary waveform (via F2F-frequency/dual-frequency modulation or modified MFM frequency) depends on the changing parameters of the medium in which the vibrations propagate, including propagation obstructions. The obtained results were mainly influenced by: the type of mechanical structure (its stiffness), the frequency of induction characteristic for any of the structural elements, and material defects such as pores or cracks. The resulting sequence of acoustic events was characteristic of each object and depended on the energy supplied to the object. This relationship was referred to as the Structural Stiffness Code (CSS). The use of this relationship and the ability to correctly interpret the received binary signal enabled the operation of the vibration controller in a wide frequency range, thanks to which it was possible to move the object with a specific algorithm.

**Figure 4:**
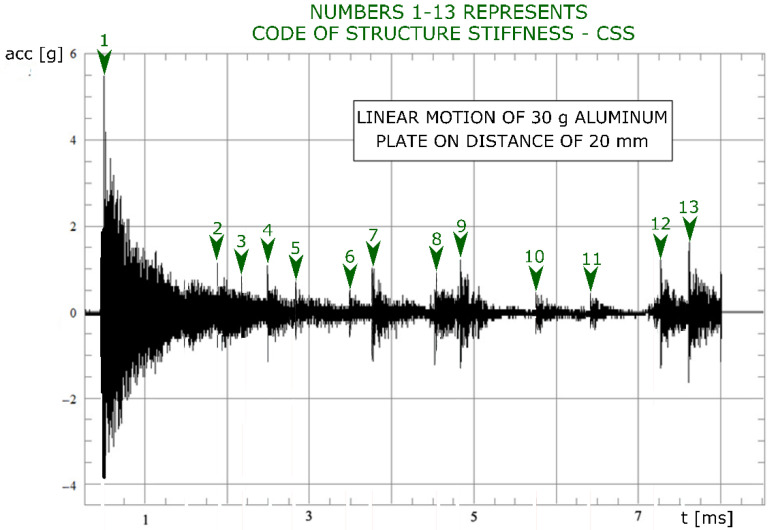
Decoded signals from ICP sensor, where the numbers from 1 to 13 represent code of the structure stiffness (CCS).

Figure 4 shows the application of the CSS method. As a result of moving the object along with the structure, it was possible to isolate several acoustic events in the signal of the ICP sensor. The essence of the CSS method is the determination of a binary series based on the duration of acoustic events and the appropriate energy released into the system by a moving object. The main influence on the amount of released energy is the period in which a high level is maintained from the initiation of a given event. In this way, a unique code connected with the state of the structure can be obtained. Work on this code and its use are the subjects of further work.

The number of acoustic events is characteristic of a specific state of the structure. Thanks to the use of a vibration regulator in a wide frequency range, it is possible to determine a specific structure “code” and to determine the dynamic characteristics of a mechanical structure. To accomplish this task, it is necessary to use a programmable pure sinusoidal excitation.

This issue requires further research, but, even at this stage, according to the authors, it can be stated that it was possible to develop a unique method that allows for the positioning of objects and the assessment of their structure/condition. The measurement technique based on the proposed solution can be an alternative to vibroacoustics and can potentially be used in SHM (Structural Health Monitoring).

### 3.4. Power and Data Transmission

In previous research by the authors, it turned out that SMART magnetic materials can be effectively used for wireless transmission of power and information [38]. The achieved results also indicated the high effectiveness of this method. The system that allowed for simultaneous data and power transmission was developed by the authors and is called SURPS (SMART Ultrasonic Resonant Power System). This system provides transmission through various solids, as well as through liquids. An additional advantage of the system is the possibility of using various transmitter–receiver configurations [38].

**Figure 5:**
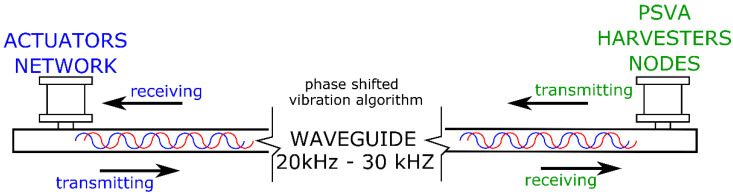
Power transmission through ultrasonic vibration—scheme.

The results presented in this paper are a continuation of work on the use of the magnetomechanical effect in the case of energy harvesting which was more widely described in [38]. As was shown earlier (Figure 2), four actuators were used instead of one. This was due to the desire to check whether it was possible to transmit energy continuously during data transmission. For this purpose, the Phase Shift Vibration Algorithm (PSVA) was used, which is schematically presented in Figure 5. In the case of this algorithm, the vibrations caused by the actuators were out of phase with each other, while each of the harvesters received a signal with a specific phase.

The transmission was carried out by an actuator that transmits mechanical energy in the form of a pure sinusoidal ultrasonic wave and then this wave was picked up by the harvester, which converted this wave into an electric current using the magneto- or electrostrictive material contained therein. This way of transmitting energy also allowed information to be sent. What is more, it was possible to send energy through different materials, and the choice of material depended mainly on the distance the energy needed to be sent.

**Figure 6:**
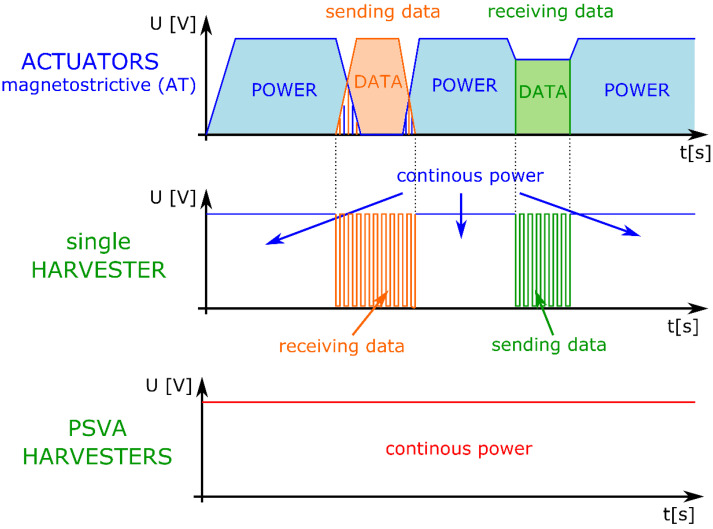
Data transmission and receiving information for PSVA.

To transmit the information, the F2F procedure was used, which is a type of frequency modulation. The modulation worked in such a way that the data transmission frequency was one order lower than the resonant frequency of the structure. Figure 6 shows, schematically, how the data were transmitted via a magnetostrictive (AT) actuator and how the signal was received on the harvester with a magnetostrictive core. In the case of using a single harvester, it is visible that there was a break in the collection of energy while receiving the signal, which means that the harvester was not powered at the moment. In the case of using more harvesters, this was transferred to each of them. The use of PSVA allowed the creation of a dedicated circuit in which each harvester received the signal in the appropriate phase so that, when data were sent to one of the harvesters, others were still powered continuously.

### 3.5. System Structure

**Figure 7:**
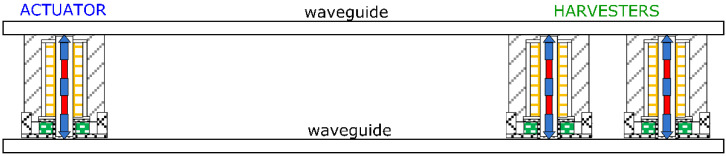
Actuator–harvester magnetostrictive system based on two beams.

The SURPS was designed to work with various actuator–harvester configurations. One such configuration is a system where harvesters are connected in series and are located between two parallel beams. Such a system is characterized by a resonance frequency above 20 kHz. The test stand, consisting of two steel rails with magnetostrictive transducers between them, is shown in Figure 7. This system made it possible to simultaneously power the microprocessor on the side of the energy harvesters and transmit data in both directions.

**Figure 8:**
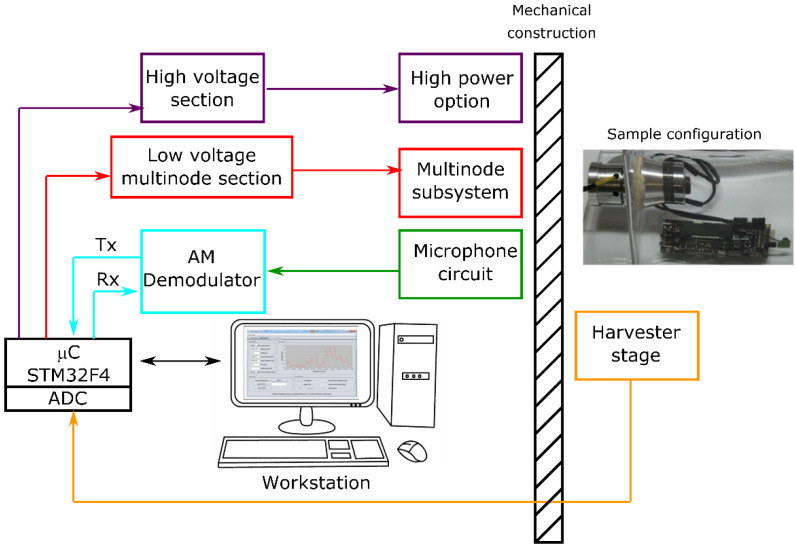
A block diagram of SURPS structure.

Based on the above-described solution, supplemented with the current state of knowledge in the field of ultrasonic techniques and wireless power transmission, an original and innovative transmitting–receiving system was developed, equipped with a microcontroller, frequency modulators, and dedicated proprietary software. Figure 8 shows a schematic overview of how the system works. The developed system has several variants, depending on what medium is used for data and energy transmission. More information on the tested system can be found in [3].

The main applications of SURPS encompass:

- service of piezoelectric or magnetic actuators/harvesters;
- scanning of a given frequency range using an actuator–harvester system with a real-time performance readout;
- searching for and generation of the resonance frequency of mechanical constructions;
- data transmission between the actuator and the harvester section in both directions (Tx, Rx), possible vibration generation in the frequency range from 0.1 Hz to 50 kHz with every 0.1 Hz (DDS generator by Analog Devices AD9851 was used);
- possible signal generation for two actuators generating vibration of the same frequency but shifted to each other in phase;
- harvester readout of current RMS voltage.

**Figure 9:**
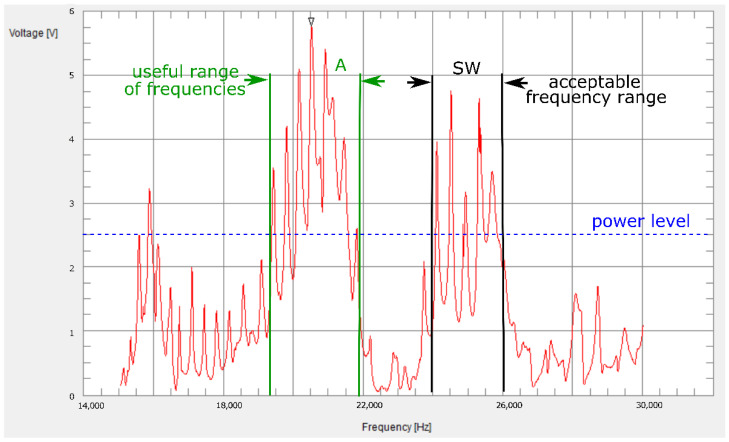
Frequency response of the dual-beam system.

The results showing the frequency-amplitude characteristics for the circuit shown in Figure 7 are presented in Figure 9. It is worth noting that the highest voltage value (the highest efficiency) was achieved for the frequency located in the above acoustic band (above 20 kHz). The zone defined as SW in the figure defines the acceptable range of resonance frequencies and is approximately 20 kHz. The dashed line shows the voltage value at the level of 2.5 V, above which loss of the microprocessor system occurred. In addition, point A mark the most advantageous frequency ranges in the case of broadcasting information. As can be seen, several frequency ranges could be distinguished that allow the system to be powered and, depending on the conditions and needs, choices could be made between the required ranges of carrier signals. It should also be mentioned that it was possible to connect more microprocessors to the harvester network, but, in that case, it was necessary to follow a strictly defined sequence of their activation. This solution allows the system to be used in SHM (Structural Health Monitoring) applications with numerous sensors.

The system was tested using a Gecko microprocessor chip with a 32-bit Cortex-M3 EFM32TG840 processor from Energy Micro. The solution for energy and information transmission proposed in the paper had to allow for powering the Gecko system, as it is a typical system used in industry. Additionally, the proposed system had to ensure a simultaneous half-duplex transmission. This meant that data were sent in one direction and at a certain time but in a two-way channel. The processor software was developed by the authors of the paper and supplemented with numerous useful functions.

**Figure 10:**
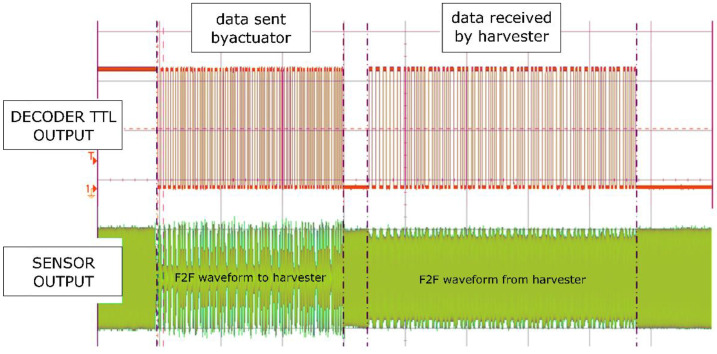
Graphic presentation of the results of power and data transfer.

The F2F-AM algorithm was used for data transmission. This algorithm guaranteed the stability of the information flow and allowed the system to achieve the transmission value at the level of 1 kbps, which was a sufficient speed in the analyzed case. To obtain higher bit rates, other frequency modulations had to be used. Figure 10 shows the results achieved for the test stand shown in Figure 7. The signal was transmitted by a magnetostrictive actuator, and the nature of the signal was sinusoidal with slight harmonic distortions. The frequency transmitted by this actuator was modulated in the on–off mode, which means that the actuator was turned on and off, which caused temporary shortages in the power supply of the energy harvester. To ensure continuous operation of the microprocessor on the harvester’s side, the system was equipped with a set of capacitors, the capacity of which was sufficient for 0.5 s of microprocessor operation. Thanks to this solution, it was possible to continuously power the microprocessor despite the temporary shortages of power supplied by the harvester and to transmit many bits of data encoded in ASCII.

The results obtained during the research showed that the developed proprietary SURPS enables the transmission of energy over distances up to 6 m without the need for wires and using only various types of mechanical structure. This solution allows the use of various types of harvester in many configurations, while the selection of the appropriate harvester system is influenced by the material and form of the medium through which energy and data are transmitted, as well as the ultrasonic wavelength.

To obtain the highest possible efficiency of both information and energy transmission while maintaining a low level of generated noise, the original software was developed. This software allows for the selection of an appropriate actuator type for a given design, as well as modulation and the recommended frequency band, thanks to which it is possible to precisely determine the values of resonance frequencies. Finally, the software selects the resonance range in which the transmission is most effective.

## 4. Conclusions

The paper presents the results of works devoted to the transmission of energy and information through various centers. The results achieved include the following:

- A prototype of a multiphase head developed based on four magnetostrictive actuators in a symmetrical system integrated with the accelerometer;
- The possibility of setting objects on the surface of the structure in motion by shaping the appropriate form of vibrations with the aid of the developed head;
- A SURPS allowing the transmission of power and information in long rods using ultrasonic vibration;
- The use of harvesters/actuators based on both magnetostrictive and piezoelectric material and the use of F2F (frequency/double-frequency) procedures, which are a type of frequency modulation, for the transmission of information;
- The development of original software to select the appropriate actuator and modulation type and recommended frequency band for energy and data transfer.

The results presented in this paper are current and constitute the basis for further work in the field of energy and data transmission. One of the main scopes for future research is the usage of controlled vibrations made by the designed actuator in NDE diagnostics. The presented method can be considered as an expansion of the methods known as energy harvesting due to the possibility for it to transform various forms of energy supplied to/collected from the system to perform mechanical work, e.g., object positioning.

## Data Availability

Data supporting reported results can be provided upon request. Currently, these data are collected as part of the ongoing project and only after its completion will the data be made available to the public.
